# Determination of Aortic Characteristic Impedance and Total Arterial Compliance From Regional Pulse Wave Velocities Using Machine Learning: An *in-silico* Study

**DOI:** 10.3389/fbioe.2021.649866

**Published:** 2021-05-13

**Authors:** Vasiliki Bikia, Georgios Rovas, Stamatia Pagoulatou, Nikolaos Stergiopulos

**Affiliations:** Laboratory of Hemodynamics and Cardiovascular Technology, Institute of Bioengineering, Swiss Federal Institute of Technology, Lausanne, Switzerland

**Keywords:** non-invasive monitoring, aorta, arterial stiffness, vascular aging, machine learning

## Abstract

*In-vivo* assessment of aortic characteristic impedance (Z_*ao*_) and total arterial compliance (C_*T*_) has been hampered by the need for either invasive or inconvenient and expensive methods to access simultaneous recordings of aortic pressure and flow, wall thickness, and cross-sectional area. In contrast, regional pulse wave velocity (PWV) measurements are non-invasive and clinically available. In this study, we present a non-invasive method for estimating Z_*ao*_ and C_*T*_ using cuff pressure, carotid-femoral PWV (cfPWV), and carotid-radial PWV (crPWV). Regression analysis is employed for both Z_*ao*_ and C_*T*_. The regressors are trained and tested using a pool of virtual subjects (*n* = 3,818) generated from a previously validated *in-silico* model. Predictions achieved an accuracy of 7.40%, *r* = 0.90, and 6.26%, *r* = 0.95, for Z_*ao*_, and C_*T*_, respectively. The proposed approach constitutes a step forward to non-invasive screening of elastic vascular properties in humans by exploiting easily obtained measurements. This study could introduce a valuable tool for assessing arterial stiffness reducing the cost and the complexity of the required measuring techniques. Further clinical studies are required to validate the method *in-vivo*.

## Introduction

Aging and vascular pathologies lead to changes in the elastic properties and the hemodynamics of the arterial network (Laurent et al., 2001; Mitchell et al., 2010; Vlachopoulos et al., 2010; Redheuil et al., 2011). These changes have been shown to be highly associated with increased cardiovascular risk or mortality (Mitchell et al., 2010; Vlachopoulos et al., 2010). In this respect, the assessment of the arterial stiffness is increasingly used in the clinical evaluation of a patient. Proximal aortic characteristic impedance (Z_*ao*_) and total arterial compliance (C_*T*_) are two powerful indices for assessing the elastic properties of the proximal aorta and the entire arterial tree, respectively (Mackenzie et al., 2002; Laurent et al., 2006).

The impedance can be defined as the ratio of the pulsatile components of pressure and flow. The impedance computed in the ascending aorta is defined as input impedance (Z_*in*_), and is a global systemic parameter, which encompasses all effects of wave travel and reflections of the arterial part which is distal to the point of measurement. For a reflectionless system Z_*in*_ reduces to Z_*ao*_. The Z_*ao*_ is a cardinal parameter related to aortic stiffness and geometry. Prior art has included invasive (Nichols et al., 1977, 1985; Pepine et al., 1978; Murgo et al., 1980; Gundel et al., 1981; Merillon et al., 1984; Ting et al., 1986; Lucas et al., 1988; Kelly and Fitchett, 1992; Kromer et al., 1992) and non-invasive (Kelly and Fitchett, 1992; Mitchell et al., 2001; Segers et al., 2007) techniques for estimating Z_*ao*_ in the frequency domain, whereby Z_*ao*_ is approximated as the average Z_*in*_ in the mid-to-high frequency range, the underlying assumption being that in those frequencies the effects of reflected waves are minimal. Other approaches have proposed time-domain calculations of the Z_*ao*_ based on the early systolic part of pressure and flow waveforms (Dujardin and Stone, 1981; Li, 1986; Levy et al., 1988; Lucas et al., 1988), when reflections are considered negligible. All of the above frequency and time domain methods require pressure and flow in the aorta, which can be obtained only invasively (pressure) or are not easy in clinical practice (flow).

C_*T*_ is a major global elastic property of the arterial system, being a determinant of the cardiac afterload, and has significant pathophysiological relevance (Safar and London, 1987; Chemla et al., 2003; Haluska, 2006; Haluska et al., 2008). It quantifies the capacity of the vessels to expand under internal pressure and store blood during systole without excessive pressure rise. Importantly, C_*T*_ is a significant determinant of central blood pressure and decrease in C_*T*_ is associated with hypertension. However, direct *in-vivo* non-invasive measurement of C_*T*_ cannot be performed. Various methods have suggested the indirect estimation of C_*T*_ (Liu et al., 1986; Segers et al., 1999; Stergiopulos et al., 1999; Mackenzie et al., 2002) using simultaneous recordings of the proximal aortic pressure wave (invasive) and flow or cardiac output.

Precise measurement of the Z_*ao*_ and C_*T*_ may increase understanding of arterial physiopathology, and provide clinical markers for cardiovascular risk and useful tools for treatment monitoring. Yet, despite the significant body of research, the invasive nature or/and the complexity of the current methods have limited their applicability in every day clinical practice, while other surrogates of regional arterial stiffness have been used more often (Mackenzie et al., 2002; Sakuragi and Abhayaratna, 2010). Thus, a technique that offers a reliable, non-invasive, fast, and simple-to-use estimation of Z_*ao*_ and C_*T*_ is still highly desirable. In view of this need, this study proposes a novel methodology to evaluate Z_*ao*_ and C_*T*_ using machine learning (ML).

**FIGURE 1:**
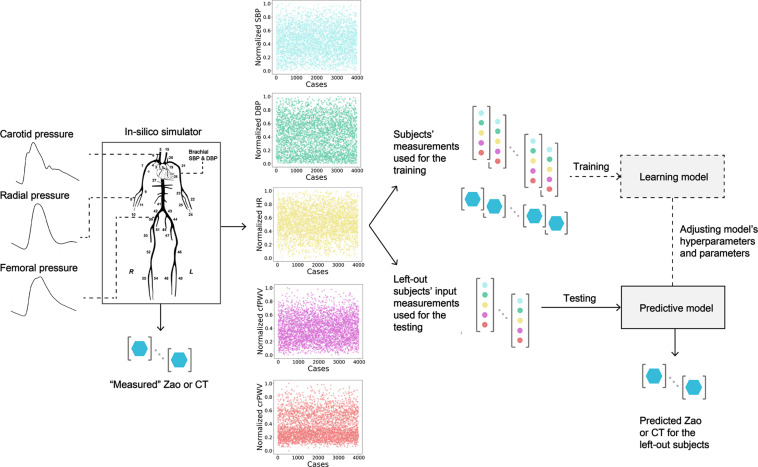
Schematic representation of the training/testing pipeline for predicting aortic characteristic impedance (Z_*ao*_), and total arterial compliance (C_*T*_). SBP, systolic blood pressure; DBP, diastolic blood pressure; HR, heart rate; cfPWV, carotid-femoral pulse wave velocity; crPWV, carotid-radial pulse wave velocity.

In our previous work, we demonstrated that the combination of *in-silico* data with ML modeling allows for validating a methodology for predicting aortic hemodynamics and cardiac contractility (Bikia et al., 2020b). This approach can be easily extended and adapted in the estimation of different cardiovascular quantities and case studies, such the one introduced in this work. Concretely, this paper proposes a method which derives Z_*ao*_ and C_*T*_ from brachial blood pressure (cuff BP) and regional PWV measurements, while it does not require central BP or flow values. To assess the validity of this concept, the introduced methodology was tested using an *in-silico* population generated by a previously validated cardiovascular simulator. The schematic representation of the regression pipeline is illustrated in Figure 1.

## Materials and Methods

### *In-silico* Database

**FIGURE 2:**
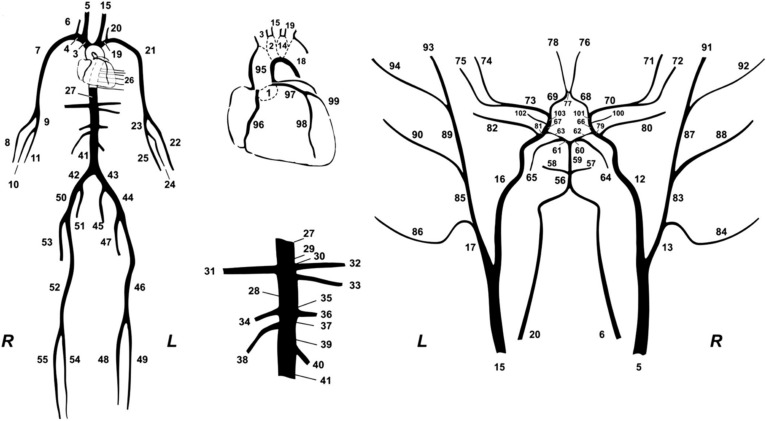
The 1-D cardiovascular model (Reymond et al., 2009) that was used for the data generation.

In this study, we used a synthetic database which was designed to simulate various hemodynamical states. Different hemodynamic cases (*n* = 3,818), representing both normotensive and hypertensive adults, were simulated by altering key cardiac and systemic parameters of a previously validated *in-silico* cardiovascular model. The mathematical model (Figure 2) has been well described in the original publication (Reymond et al., 2009). Literature data are only presented in terms of mean and standard deviation or/and minimum and maximum values; thus, variation of the model’s parameters was performed with random Gaussian sampling. Cardiac parameters were modified and different cardiac output values were simulated. Arterial geometry (i.e., arterial length and diameter) was modified to represent various arterial tree sizes and body types (Wolak et al., 2008; Devereux et al., 2012). Total peripheral resistance and arterial compliance were altered according to the literature (Langewouters, 1982; Lu and Mukkamala, 2006; Segers et al., 2008). To simulate older or hypertensive individuals, in some cases, stiffening in the aorta was considered as non-uniform and more pronounced as described in our previous works (Pagoulatou et al., 2019; Bikia et al., 2020a). For a given set of input parameters, the model provides analytical solutions of the pressure and flow at every arterial segment. The physiological validity of each subject was assessed by comparing the simulated brachial and aortic systolic BP (SBP), DBP, MAP, and pulse pressure (PP) to the reference values reported in the previously published data by McEniery (McEniery et al., 2005) (normotensive cases) and Bordin Pelazza and Filho (Bordin Pelazza and Filho, 2017) (hypertensive cases). A subject was removed from the dataset if any of the BP values lied out of the 99.5% confidence intervals (mean ± 2.807 SD).

### Computation of Z_*ao*_ and C_*T*_

The characteristic impedance at the root of the ascending aorta was calculated analytically using the area compliance and the geometry of the ascending aorta, namely:


(1) $$Z_{a\,o}=\sqrt{\frac{\rho }{A}\,\frac{1}{C_{A}}},$$

where ρ is the blood density equal to 1,050 kg/m^3^, A is the cross-sectional area of the ascending aorta, and C_*A*_ is the area compliance of the ascending aorta, respectively.

The C_*T*_ was computed as the sum of volume compliance (c_*i*_) of all the arterial segments (*n* = 103) included in the 1-D model and the terminal compliances described by the terminal Windkessel models, namely:


(2) $${\text{C}}_{\text{T}}=\sum_{\text{i}}^{103}{\text{c}}_{\text{i}}$$

### Regional PWVs and BP Data

The carotid-femoral pulse wave velocity (cfPWV) and carotid-radial pulse wave velocity (crPWV) were calculated by a foot-to-foot algorithm using the tangential method (Vardoulis et al., 2013). Pulse transit times were computed between the two arterial sites, namely, the left carotid and left femoral artery, and the left carotid and the left radial artery, respectively. Formally, the tangential method uses the intersection point of two tangents on the arterial pressure wave, i.e., the tangent passing through the systolic upstroke and the horizontal line passing through the minimum of the pressure wave as previously described (Vardoulis et al., 2013). The travel lengths were determined by summation of the lengths of the arterial segments within the transmission paths. Next, the value of each PWV was calculated by dividing the total travel length by the pulse transit time. Brachial systolic (brSBP) and diastolic BP (brDBP) were derived from the pressure waveform at the left brachial artery.

### Regression Analysis

The simulated data, i.e., brSBP, brDBP, heart rate (HR), cfPWV, crPWV, Z_*ao*_, and C_*T*_, were organized in pairs (inputs: brSBP, brDBP, HR, cfPWV, crPWV, and outputs: Z_*ao*_, C_*T*_) and were kept for the training/testing process. All the data were corrupted with artificial noise in order to simulate potential measurement errors that often occur in the respective clinical measurements. The noise allows for essentially harming the deterministic effect of the 1-D computer model. Errors in measurements were simulated with a random distribution. Concretely, the error for each variable was randomly drawn from the range of [–15, 15]% (simulating a maximum noise level equal to ± 15%). Subsequently, each variable value was multiplied with a noise factor; for instance, for a randomly selected error of –6%, the respective variable value was multiplied with a noise factor equal to 0.94.

The data were partitioned into three subsets: (i) the train set, the set of training examples the model is trained on, (ii) the validation set, which is used to tune the hyperparameters, and (iii) the test set, which is used to test the trained model while it measures the generalization performance. In our analysis, the train/validation/test split was selected to be 60/20/20%. These percentages corresponded to 2,290/764/764 data instances, respectively. The train and test sets were normalized using MinMaxScaler() function from sklearn library. For the regression process, we used a Random Forest modeling procedure (Liaw and Wiener, 2002) and an Artificial Neural Network (ANN) to estimate the target variables of interest.

**FIGURE 3:**
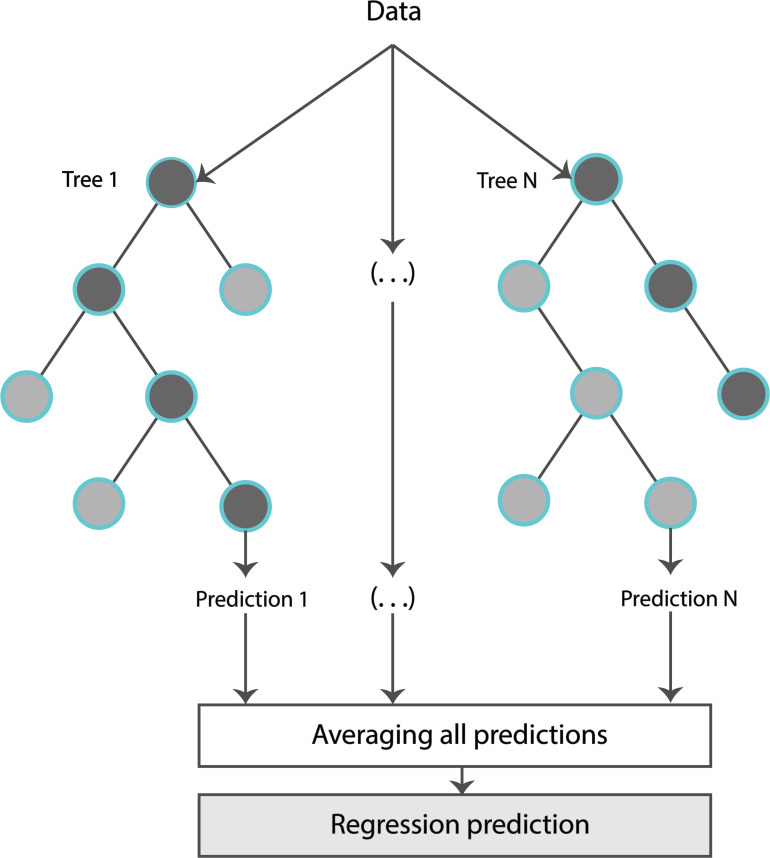
Typical representation of a random forest regressor.


The formal structure of the Random Forest Regressor (RFR) is shown in Figure 3. Concretely, an RFR is a predictor consisting of a collection of randomized base regression trees. These random trees are combined to form the aggregated regression estimate

$${\bar{r}}_{n}\,\left(X\right)=E_{\Theta }\,\left[r_{n}\,\left(X,\Theta \right)\right],$$

where E_Θ_[.] denotes expectation with respect to the random parameter, conditionally on **X** (matrix consisting of the input features), and Θ = [Θ1, …, ΘN] are independent and identically distributed (i.i.d.) random variables outputs of each tree. The estimations were provided by aggregating the individual predictions of each tree. The trees were grown by applying bootstrapping. Based on the training data, each regression tree grew for each of the bootstrap samples. Estimators were randomly sampled and the best split was chosen at each node.

**FIGURE 4:**
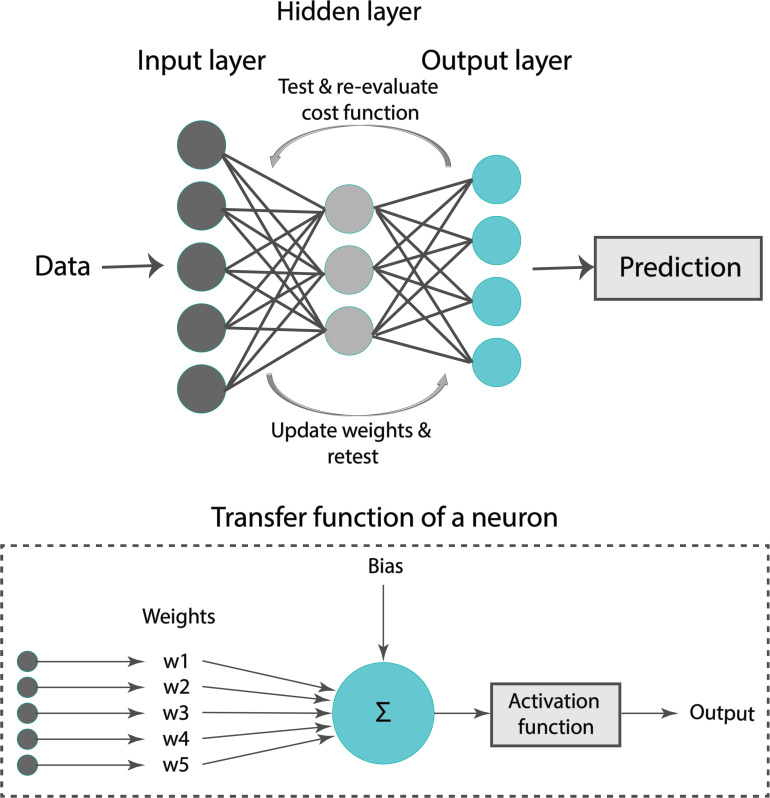
Typical representation of an artificial neural network.

A formal representation of an ANN is illustrated in Figure 4. Our ANN was composed of an input layer, a hidden layer, and an output layer. Typically, the input layer sequentially receives the input features as an input vector into the ANN. The hidden layer has multiple neurons connected to the input layer with weights. Each neuron is characterized by a transfer function of neuron (Figure 4). The training of ANN is conducted by determining the difference between the processed output of the network and the target output, namely, the error. Training data are fed to the input layer and continue to the succeeding hidden layer, where they pass through the neurons’ transfer functions, until they finally arrive radically transformed at the output layer. During training, the network continually adjusts its weights and thresholds until the ANN produces output which is increasingly similar to the target output (errors are minimized). In our analysis, the training set was employed to optimize the weights of neurons in the hidden and output layer using the “Adam” optimizer (Kingma and Ba, 2017). Upon tuning, the samples of the test set were used as input to the optimized ANN to obtain the estimated Z_*ao*_ and C_*T*_.

A critical issue while training a ML model on the sample data is overfitting. For instance, when the number of epochs used to train an ANN is more than necessary, the training model learns patterns that are specific to the sample data to a great extent. In that case, the model is incapable to perform well on a new dataset. In other words, the model loses generalization capacity by overfitting to the training data. To mitigate overfitting and to increase the generalization capacity, the model should be trained for optimal hyperparameter values.

For the RFR, we selected 100 *estimators* (namely, the number of trees in the forest), while we decided to optimize the value for *max_depth* (the maximum depth of the tree). For the ANN, the *batch_size* (the number of samples that will be propagated through the network) was set to be equal to 10, and the number of *epochs* was optimized, respectively. The number of *epochs* is a hyperparameter that defines the number of times that the learning algorithm will work through the entire training dataset. By optimizing only one hyperparameter, we keep the complexity of the models low, and thus the models are more likely to perform well on new data and are less restricted to the peculiarities of the particular data used.

**FIGURE 5:**
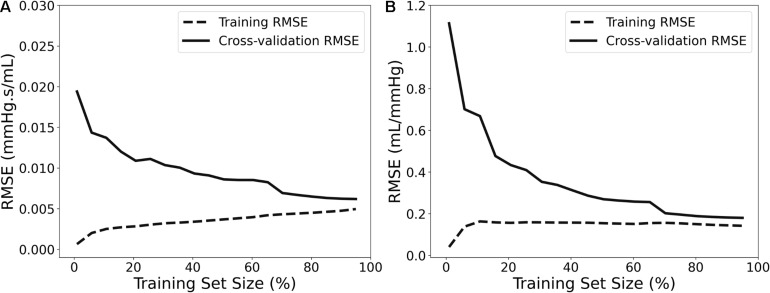
Learning curves presenting the impact of the number of data instances on the RFR’s performance for Z_*ao*_ **(A)** and C_*T*_ **(B)**.

For selecting the optimal value for *max_depth*, we calculated the train score and the validation score for various values of *max_depth* in the range of [1, 10]. The score for RFR indicates the coefficient of determination R^2^ for the predictions. Subsequently, for each target output variable, the *max_depth* value with the maximum score was selected. In a similar manner, the train and validation losses [i.e., mean square error (MSE)] were calculated for the ANN. In that case, loss values can be monitored by Early stopping call back function. When there is an increment observed in loss values, training comes to halt and the respective value of epoch indicates the optimal selection. For both Z_*ao*_ and C_*T*_, the highest accuracy was reported for the RFR with *max_depth* = 8, whereas for ANN, training stopped at 55th epoch and 103rd epoch for Z_*ao*_ and C_*T*_, respectively. Therefore, the optimal number of epochs was set to 55 and 103, for the two estimators, respectively. All optimized hyperparameters are aggregated in Table 1. Subsequently, we plotted the respective learning curves for the RFRs using the optimal hyperparameters (Figures 5A,B). Each learning curve was fitted using the observed accuracy [in terms of root mean square error (RMSE)] according to a given training sample size. The training size was modified from 1 to 95% of the total number of training data instances (20 samples of training size). The training error was low, and thus the training data appear to fit well by the models (low bias). Furthermore, low variance was indicated by the small gap between the two curves. Finally, the testing set was fed into the trained RFR to estimate Z_*ao*_, and C_*T*_ and the precision was evaluated.

**TABLE 1:** List of the selected hyperparameters for the predictive models.

Output variable	Selected hyperparameters	
	*RFR: max_depth*	*ANN: epochs*
Z_*ao*_	8	55
C_*T*_	8	103

*RFR, Random Forest Regressor; Z_ao_, aortic characteristic impedance; C_T_, total arterial compliance.*

Along with the main model configuration, which uses as inputs brSBP, brDBP, HR, cfPWV, and crPWV (M1), we additionally evaluated three additional model configurations using different sets of inputs: (i) one which does not include HR as an input (M2), (ii) a second one that excludes HR and replaces brSBP and brDBP with MAP (M3), and (iii) a third one that uses only the PWV values (M4). The hyperparameters values were set equal to the same values as those of M1.

Furthermore, we assessed the importance of each input feature using the permutation feature importances (Breiman, 2001) for RFR. The concept of permutation feature importances relies on measuring the importance of a feature by calculating the increase in the prediction error after permuting the feature. The permutation importances were computed by shuffling the values of each feature on the test set and by estimating the RMSE after the permutation. This process was repeated 20 times and the mean and standard deviation of the increase in RMSE were reported.

The training/testing pipeline and the post-analysis were implemented using the Scikit-learn library (Pedregosa et al., 2011) in a Python programming environment (Python Software Foundation, Python Language Reference, version 3.6.8, Available at http://www.python.org). The Pandas and NumPy packages were also used (Oliphant, 2006; McKinney, 2010).

### Sensitivity to Training Data Size

The number of data instances used for training, namely, the training size, has a major effect on the accuracy of the model’s predictions. The model’s precision as a function of the number of training samples was evaluated by conducting sensitivity analysis. In this respect, the regression analysis for RFR was repeated and the RMSE was calculated after decreasing the training size (*n* = 2,290) from 99 to 1% of the total number of cases. The accuracy was assessed using the same testing population (764 subjects).

### Comparison to Prior Art

We compared our RFR with prior methods that provide estimates of Z_*ao*_ and C_*T*_. Application of previous methods required the central (aortic) blood pressure and flow waves. Systolic and diastolic phases were defined by the dicrotic notch from the central blood pressure waveforms and the first zero crossing for blood flow waves. Automated detection of the peaks and minima was performed using an in-house custom software in Matlab (Mathworks, Natick, Massachusetts, United States). The Z_*ao*_ was computed using two previously described methods. For this study, we used the following formulas:

1. *Time-derivative peaks method*: Z_*ao*_ = P’_*max*_/Q’_*max*_, where P’_*max*_ and Q’_*max*_ are the maximum values of the pressure and flow time derivatives, respectively.
2. *Peak flow method*: Z_*ao*_ = (P_*Qmax*_–aDBP)/Q_*max*_, where aDBP is the aortic DBP, Q_*max*_ is the maximum flow value, and P_*Qmax*_ is the aortic pressure magnitude at the maximum flow value.

The C_*T*_ was derived using the following previously proposed techniques:

1. *Decay time method:* The decay time method (DTM) is based on the two-element Windkessel (WK) model of the systemic circulation. Its principle is that during diastole there is no inflow from the heart, and thus, the decrease of aortic pressure, is characterized by the decay time. This decay can be fitted monoexponentially to any portion of the diastole to yield the characteristic time or time constant, which is RC_*T*_. The C_*T*_ can be then calculated for a known value of peripheral resistance (R) (Stergiopulos et al., 1995).
2. *Pulse pressure method*: The pulse pressure method (PPM) (Stergiopulos et al., 1994) is based on the fact that the modulus of the input impedance of the arterial system is represented very well by the two-element WK model for the low frequencies (1st–5th harmonic). Therefore, the pulse pressure will be similar in the true arterial system and the two-element WK model. The PPM uses an iterative process that yields the value of C_*T*_ that gives the best fit between the measured pulse pressure and the pulse pressure predicted by the 2-element WK model.

We applied the aforementioned methods on the test data (*n* = 764) and compared the estimates to the ML-derived predictions. The reason that we did not apply the above methods to the entire dataset was to compare these methods and the ML model on the exact same test population. Artificial random noise of the same order of magnitude (±15%) was also considered for the data used for the techniques above. The pressure and flow signals were uniformly multiplied by a scaling factor which was randomly selected as described in the Regression analysis section.

### Statistical Analysis

Data are presented as mean and standard deviation (SD). The agreement, bias, and precision between the model predictions and the reference values were assessed by using the Pearson’s correlation coefficient (r), the RMSE, the normalized RMSE, and the Bland-Altman analysis (Bland and Altman, 1986). The nRMSE was based on the difference between the minimum and maximum values of the dependent variable (y) and was computed as RMSE/(y_*max*_–y_*min*_). We performed linear least-squares regression for the predictions and the reference data. The slope and the intercept of the regression line were reported. Two-sided p-value for a hypothesis test whose null hypothesis is that the slope is zero, using Wald Test with t-distribution of the test statistic, was calculated. The *p* < 0.05 were considered as significant. The statistical analysis was implemented in Python (Python Software Foundation, Python Language Reference, version 3.6.8, Available at http://www.python.org).

## Results

The distributions of the cardiovascular parameters of the virtual study cohort are presented in Table 2. The correlations between the input features and the target output values are also given in Table 3. The highest values of Pearson’s correlation coefficient were reported between Z_*ao*_/C_*T*_ and the two PWV values (*r* ≥ 0.84).

**TABLE 2:** Summary of the virtual study cohort (*n* = 3,818) cardiovascular characteristics.

Variable	Mean ± SD
	Entire population *n* = 3,818
Brachial SBP [mmHg]	134.51 ± 24.1
Brachial DBP [mmHg]	77.27 ± 21.31
Brachial PP [mmHg]	57.24 ± 22.58
MAP [mmHg]	94.51 ± 20.29
Aortic SBP [mmHg]	122.54 ± 23.73
Aortic DBP [mmHg]	80.5 ± 21.48
Aortic PP [mmHg]	42.04 ± 19.38
Stroke volume [mL]	81.18 ± 8.03
Heart rate [bpm]	73.26 ± 14.9
Aortic impedance [mmHg.s/mL]	0.056 ± 0.012
Total arterial compliance [mL/mmHg]	1.14 ± 0.47
Total peripheral resistance [mmHg.s/mL]	0.98 ± 0.21
Carotid-femoral PWV [m/s]	8.06 ± 1.03
Carotid-radial PWV [m/s]	10.17 ± 1.3

*SD, standard deviation; SBP, systolic blood pressure; DBP, diastolic blood pressure; PP, pulse pressure; MAP, mean arterial pressure; PWV, pulse wave velocity.*

**TABLE 3:** Correlation coefficients between the input regression features and the target outputs.

Parameters	Value (*n* = 3,818)
	Correlation coefficient
brSBP/Z_*ao*_	0.51
brDBP/Z_*ao*_	–0.41
HR/Z_*ao*_	0.17
cfPWV/Z_*ao*_	**0.87**
crPWV/Z_*ao*_	**0.85**
brSBP/C_*T*_	–0.48
brDBP/C_*T*_	0.39
HR/C_*T*_	–0.16
cfPWV/C_*T*_	−**0.87**
crPWV/C_*T*_	−**0.84**

*Highest correlation values are presented in bold. brSBP, brachial systolic blood pressure; brDBP, brachial diastolic blood pressure; HR, heart rate; PWV, pulse wave velocity; cfPWV, carotid-femoral PWV; crPWV, carotid-radial PWV; Z_ao_, aortic characteristic impedance; C_T_, total arterial compliance.*

### Comparison of Model Predictions to the Reference Values

**FIGURE 6:**
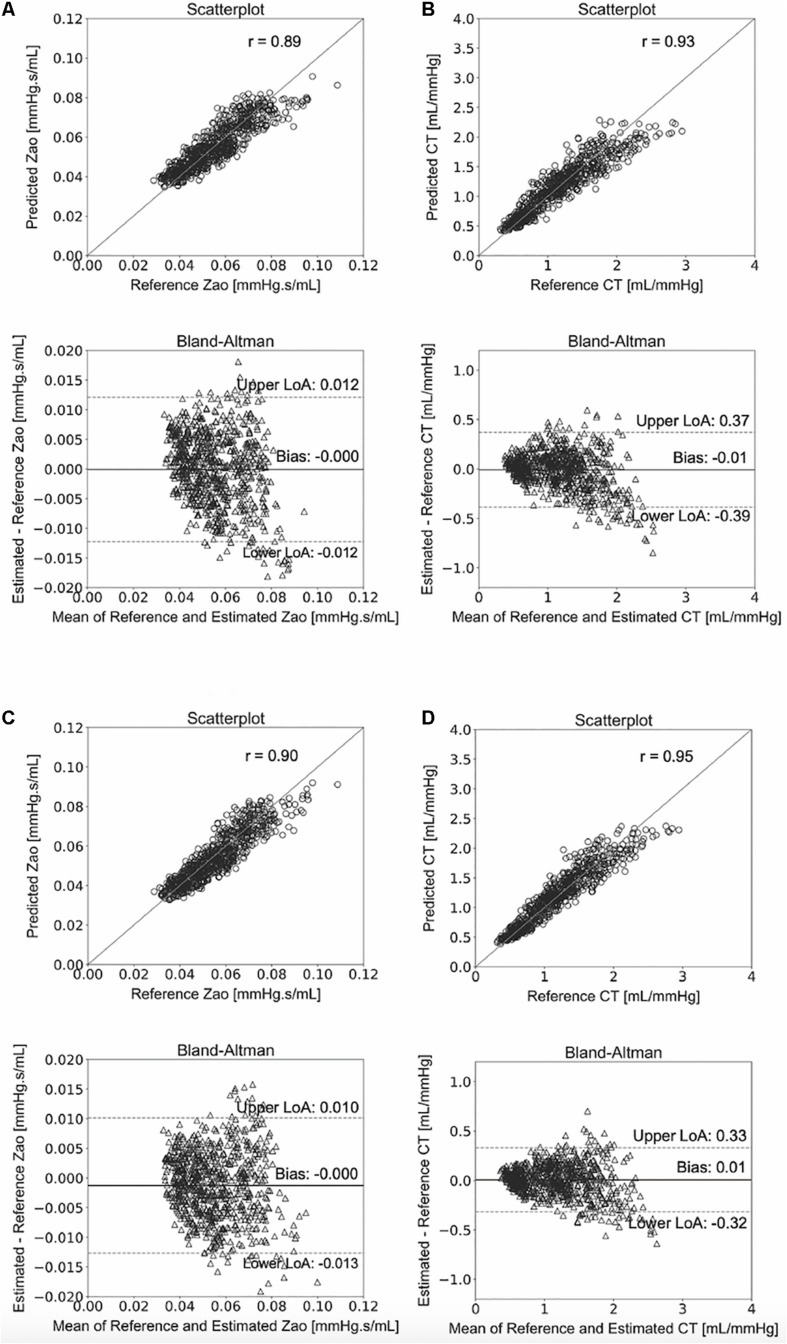
Comparison between predicted and reference data. Scatterplots and Bland–Altman plots between: the predicted Z_*ao*_ and the reference Z_*ao*_ using the RFR **(A)** and the ANN **(C)**, respectively, and the predicted C_*T*_ and the reference C_*T*_ using the RFR **(B)** and the ANN **(D)**, respectively. The solid line of the scatterplots represents equality. In Bland–Altman plots, limits of agreement (LoA) are defined by the two horizontal dashed lines.

We compared the RFR and ANN estimations to the reference data for each target output. Table 4 tabulates the metrics for the performance assessment of the evaluation scheme for all model configurations. The results for the RFR M1 and ANN M1, which correspond to the best-performing configurations, are visualized below. The scatterplot between the RFR-predicted and the actual Z_*ao*_ values are given in Figure 6A (top panel). The Bland-Altman plot is provided in Figure 6A (lower panel), in which zero bias was reported. The limits of agreement (LoA), within which 95% of errors are expected to lie, were found to be equal to ± 0.012 mmHg.s/mL. Figure 6B illustrates the C_*T*_ predictions in comparison to their reference values. Again, bias was close to zero (–0.01 mL/mHg), while the LoA were equal to ± 0.4 mL/mmHg. The scatterplot and Bland-Altman plot for the ANN are shown in Figures 6C,D for Z_*ao*_ and C_*T*_, respectively. For Z_*ao*_, the ANN-LoA were [–0.013, 0.010] mmHg.s/mL, whereas for C_*T*_ predictions, the ANN-LoA found to be subtly narrower than the RFR and equal to ± 0.3 mL/mmHg. For both ML approaches, no biases were reported. The mean difference between the Z_*ao*_ predictions and the ground truth Z_*ao*_ values lied within a similar range for the two models, i.e., [–0.012, 0.012] and [–0.013, 0.010] mmHg.s/mL for RFR and ANN, respectively. The LoA of the C_*T*_-RFR ([–0.39 0.37] mL/mmHg) were slightly broader than the LoA of the C_*T*_-ANN estimator ([–0.32 0.33] mL/mmHg). Substantially higher errors were reported when the BP information was omitted from the inputs, especially for the Z_*ao*_ prediction (correlation was around to 0.75). Table 5 presents the feature importances of the input regressors for Z_*ao*_ and C_*T*_, respectively. For Z_*ao*_, brDBP appeared to have the highest importance level followed by brSBP and crPWV. In the case of C_*T*_, brDBP was reported to have the dominant importance value, followed by cfPWV and crPWV.

**TABLE 4:** Regression statistics between model predictions and reference values.

Model	Slope	Intercept	r	*p*-value	RMSE	nRMSE
RFR_*Zao*_ M1	0.77	0.012 mmHg.s/mL	0.89	<0.001	0.006 mmHg.s/mL	7.78%
RFR_*Zao*_ M2	0.77	0.013 mmHg.s/mL	0.89	<0.001	0.006 mmHg.s/mL	7.77%
RFR_*Zao*_ M3	0.66	0.019 mmHg.s/mL	0.81	<0.001	0.008 mmHg.s/mL	9.91%
RFR_*Zao*_ M4	0.55	0.025 mmHg.s/mL	0.75	<0.001	0.009 mmHg.s/mL	11.20%
RFR_*CT*_ M1	0.81	0.21 mL/mmHg	0.93	<0.001	0.19 mL/mmHg	7.31%
RFR_*CT*_ M2	0.80	0.22 mL/mmHg	0.93	<0.001	0.19 mL/mmHg	7.37%
RFR_*CT*_ M3	0.73	0.31 mL/mmHg	0.88	<0.001	0.24 mL/mmHg	9.21%
RFR_*CT*_ M4	0.63	0.42 mL/mmHg	0.82	<0.001	0.29 mL/mmHg	11.11%
ANN_*Zao*_ M1	0.86	0.007 mmHg.s/mL	0.90	<0.001	0.006 mmHg.s/mL	7.40%
ANN_*Zao*_ M2	0.77	0.012 mmHg.s/mL	0.90	<0.001	0.006 mmHg.s/mL	7.47%
ANN_*Zao*_ M3	0.69	0.016 mmHg.s/mL	0.83	<0.001	0.008 mmHg.s/mL	9.60%
ANN_*Zao*_ M4	0.56	0.022 mmHg.s/mL	0.76	<0.001	0.009 mmHg.s/mL	11.28%
ANN_*CT*_ M1	0.88	0.14 mL/mmHg	0.95	<0.001	0.16 mL/mmHg	6.26%
ANN_*CT*_ M2	0.89	0.17 mL/mmHg	0.94	<0.001	0.18 mL/mmHg	6.87%
ANN_*CT*_ M3	0.75	0.29 mL/mmHg	0.88	<0.001	0.24 mL/mmHg	9.29%
ANN_*CT*_ M4	0.64	0.45 mL/mmHg	0.83	<0.001	0.29 mL/mmHg	10.94%

*RMSE, root mean square error; nRMSE, normalized RMSE.*

*M1 uses as inputs brSBP, brDBP, HR, cfPWV, crPWV.*

*M2 uses as inputs brSBP, brDBP, cfPWV, crPWV.*

*M3 uses as inputs MAP, cfPWV, crPWV.*

*M4 uses as inputs cfPWV, crPWV.*

**TABLE 5:** Feature importances for the prediction of Z_*ao*_ and C_*T*_ using RFR.

Feature	Permutation importance	
	Z_*ao*_ [mmHg.s/mL]	C_*T*_ [mL/mmHg]
Brachial SBP	0.0031 ± 0.0002	0.09 ± 0.01
Brachial DBP	0.0058 ± 0.0002	0.21 ± 0.01
Heart rate	0.0001 ± 0.0000	0.01 ± 0.00
Carotid-femoral PWV	0.0016 ± 0.0001	0.13 ± 0.01
Carotid-radial PWV	0.0021 ± 0.0001	0.12 ± 0.01

*SD, standard deviation; SBP, systolic blood pressure; DBP, diastolic blood pressure; PWV, pulse wave velocity.*

### Sensitivity to Training Data Size

**FIGURE 7:**
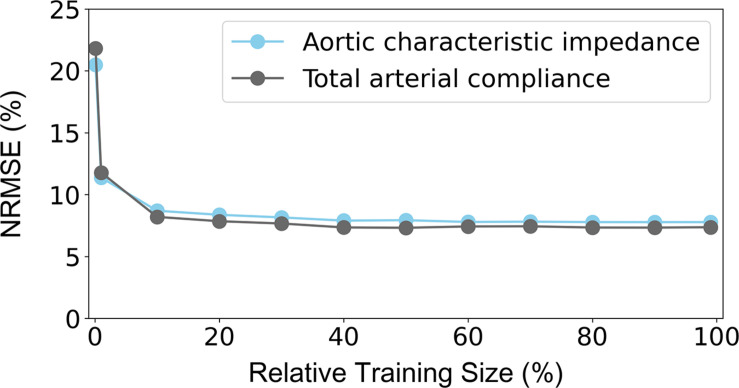
Sensitivity of precision in terms of NRMSE to the number of the training data. The 100% of the training size corresponds to 2,290 cases. NRMSE: normalized root-mean square error.

The NRMSEs decreased gradually with increasing training size (Figure 7). Errors in Z_*ao*_ were higher than 8% for a training dataset with 687 subjects or less. The NRMSE of the C_*T*_ predictions exceeded 8% when the training size was smaller than 458 data instances. It was observed that, for both curves, addition of new data points had no significant impact on the accuracy after reaching the 20% of the entire training population (corresponding to 458 subjects).

### Comparison to Prior Art

Table 6 presents the comparison between our proposed PWV-based ML models and a list of previously published methods, which, in contrast to our method, use the central aortic blood pressure and flow waveforms. The PWV-based ML estimators for Z_*ao*_ outperformed all the other methods achieving a correlation of 0.9. The peak flow method and the time-derivative peaks method demonstrated lower accuracy (*r* ≤ 0.79 and broader LoA). Estimation techniques for C_*T*_ yielded correlation coefficients equal or higher than 0.93.

**TABLE 6:** Comparison of the proposed ML-based Z_*ao*_ and C_*T*_ estimators to prior art.

Method	Estimated Z_*ao*_ (mmHg.s/mL)	Reference Z_*ao*_ (mmHg.s/mL)	Error (%)	r	Bland-altman biases (mmHg.s/mL)
Time-derivative peaks method*	0.044 ± 0.008	0.056 ± 0.014	Min: –55 Max: –20	0.66	–0.011 [–0.030, 0.007]
Peak flow method*	0.071 ± 0.018		Min: –14 Max: 107	0.79	0.016 [–0.007, 0.038]
PWV-based RFR	0.056 ± 0.013		Min: –27, Max: 34	0.89	–0.000 [–0.012, 0.012]
PWV-based ANN	0.056 ± 0.012		Min: –21, Max: 37	0.90	–0.000 [–0.013, 0.010]

	**Estimated C_*T*_ (mL/mmHg)**		**Errors (%)**		**Bland-altman biases (mL/mmHg)**

Decay time method*	1.60 ± 0.71	1.16 ± 0.51	Min: –10, Max: 152	0.93	0.44 [–0.16, 1.05]
Pulse pressure method*	1.23 ± 0.48		Min: –24, Max: 58	0.94	0.07 [–0.27, 0.41]
PWV-based RFR	1.16 ± 0.45		Min: –36, Max: 62	0.93	–0.01 [–0.39, 0.37]
PWV-based ANN	1.18 ± 0.48		Min: –28, Max: 57	0.95	0.01 [–0.32, 0.33]

*Z_ao_, aortic characteristic impedance; C_T_, total arterial compliance.*

*Errors are expressed as 100 x (X_est_–X_actual_)/X_actual_, where X is the target output under test (i.e., Z_ao_ or C_T_). Limits of agreement are defined as [mean bias + 1.96 SD, mean bias–1.96 SD].*

*These methods utilize both the central blood pressure and flow waves for estimating Z_ao_ and C_T_.

## Discussion

The Z_*ao*_ contributes to the pulsatile arterial load faced by heart during ejection and has been shown to be an independent predictor of LV mass index in hypertension (Chirinos et al., 2010). Moreover, C_*T*_ offers a valuable assessment not only for cardiovascular (CV) risk, but also for the relationship between structural and functional changes in the arterial system with respect to its elasticity (Mendonça et al., 2009; Haluska et al., 2010). In a progressively aging population, effective monitoring of powerful biomarkers, such as Z_*ao*_ and C_*T*_, is imperative. Despite the great efforts for monitoring several biomarkers for arterial stiffness, there is evidence that the prognostic value of arterial stiffness as assessed by current techniques might be compromised in the elderly or special populations (Megnien, 1998; Meaume et al., 2001; Protogerou et al., 2007; Verwoert et al., 2012). Furthermore, there are methods, such as the pulse contour techniques for minimally invasive cardiac output monitoring, which are dependent on C_*T*_ value (de Wilde et al., 2007).

Measurement of PWV can be utilized for the estimation of both local (Rabben et al., 2004; Borlotti et al., 2010) and regional arterial distensibility (Laurent et al., 2006). The evaluation of PWV is based on the estimation of the pulse transit time between two arterial sites, and the measurement of the distance between them. There is emerging evidence supporting that aortic PWV, i.e., between the carotid and femoral artery, is an independent predictor of CV risk (Sakuragi and Abhayaratna, 2010; Vlachopoulos et al., 2012). Likewise, the peripheral PWV, e.g., between the carotid and the radial artery, has been shown to be an informative indicator of vasodilator reserve and a predictor of coronary artery disease (Lee et al., 2006). Despite the widespread acceptance of PWV, we should not be detracted from the fact that PWV *per se* is still an indirect measure of arterial properties and provides no immediate measure of the adverse effects of vascular stiffening on circulatory hemodynamics. For instance, although PWV might be often clinically relevant, it is not the sole determinant of the timing and consequences of the reflected waves (Asmar et al., 2001; Williams et al., 2006). There is no doubt that C_*T*_ is physiologically more relevant than regional or local arterial compliance surrogate (such as PWVs), in terms of modulation of cardiac load, LV function, and CV risk assessment. In particular, the C_*T*_ can have greater impact in assessing elderly population or individuals with increased vascular stiffness, where PWV appears to have limited prognostic value. Moreover, Z_*ao*_ has been associated with cases of increased cardiac and cerebral mortality (Mitchell et al., 2010; Vlachopoulos et al., 2012). On the other hand, PWV is computed between two arterial sites, and thus cannot provide a global description of the arterial network as Z_*ao*_ does. Evidence reported by Segers et al. (2007) presents that measurement of central pressure and flow for the evaluation of global arterial parameters is more relevant and provides major mechanistic information that it should be also considered when the more frequently acquired PWV is evaluated.

Knowledge of Z_*ao*_ and C_*T*_ might have additional diagnostic impact as well as additive prognostic value beyond PWV. Estimation of the Z_*ao*_ and C_*T*_ is, however, difficult in clinical practice, as it requires concomitant recordings of pressure and flow waveforms in the proximal aorta (Randall et al., 1976; Lucas et al., 1988; Stergiopulos et al., 1994, 1995; Mitchell et al., 2001). The methodological complexity and lack of validation have prohibited their application in the everyday clinical practice. For this reason, capitalization of the regional PWV measurements for estimating Z_*ao*_ and C_*T*_ may permit their clinical assessment in a simple and cost-efficient way.

The present study suggested a ML predictive tool for Z_*ao*_ and C_*T*_ by using regional PWV measurements and cuff BP. The carotid-femoral PWV is a measure of central arterial stiffness, whereas the carotid-radial PWV expresses a mix of central and peripheral stiffness of the arterial tree. The principle of this concept is that the combined information embedded in the two indicators of regional elasticity can lead to a much-improved characterization of Z_*ao*_ and C_*T*_. The results indicated that the suggested framework appears to apply well over a wide range of simulated physiological conditions. The methodology was appraised by testing two different ML models which, with proper hyperparameters’ selection, achieved a similar predictive precision. This may also suggest that there is no high dependency on the nature of the ML approach, while it can provide preliminary evidence of the validity of the proposed framework.

Our methodology seems to offer a competitive advantage in comparison to prior methods. More specifically, it does not require central blood pressure and flow waves, for which gold standard measurements are invasive. The invasive nature of the central BP wave’s acquisition has been addressed either by the use of the carotid BP which is considered a good surrogate of aortic BP and can be easily acquired via tonometry, or by the use of devices that provide an approximation of the central BP wave via transformation of the radial BP wave (Weiss et al., 2012; Shoji et al., 2017). Measurement of central flow has been feasible by non-invasive techniques (e.g., Ultrasound or Magnetic Resonance Imaging) which are, however, expensive and rather dependent on operator skills. Yet, the results of this study showed that it outperformed some of the existing estimators. Previous methods for estimating Z_*ao*_ had significantly wider LoA when compared to our PWV-based ML estimators, while all current methods were also found to have high biases (>0.01 mmHg.s/mL). For C_*T*_, PWV-based ANN had a similar performance to the PPM estimator, while the DTM yielded a lower precision. It is to be stressed that the comparison of the PWV-based ML estimators with the prior art cannot be direct and absolute, due to two main reasons: (i) the different nature of the required inputs, and (ii) the simplified simulation of the measurement error in the time signals. Concretely, although the previously published techniques are non-invasive, they require simultaneous measurement of the central blood pressure and flow, which are more difficult to acquire compared to the measurement required for the proposed ML estimator. In our experiments, the testing of these methods was done using the simulated aortic blood pressure which is the gold standard; in a real clinical setting, invasive aortic blood pressure is rarely available. Regarding the noise simulation, the artificial errors to the signals were simplified; a random scaling factor was selected and multiplied with the entire signal. Hence, the error did not vary during the entire beat, and, as a result, the shape of the wave, from which the computational algorithms are highly dependent to, remained unaffected.

The main advantage of our proposed method pertains to its simplicity and convenience (for both the patient and the physician) rather than its increased accuracy in comparison to the state of the art. The existing techniques require non-invasive, yet expensive and complex, flow or velocity measurements for evaluating Z_*ao*_ and C_*T*_. It is undeniable that previous studies have shown that current non-invasive techniques provide high accuracy and reliability for both Z_*ao*_ and C_*T*_ when compared with the invasive ground truth (Kelly and Fitchett, 1992; Segers et al., 2007). However, being able to assess Z_*ao*_ and C_*T*_ from PWVs alone could be very valuable given that such an approach eliminates the need for flow measurement which requires Echocardiographic or Magnetic Resonance Imaging procedures. Undoubtedly, both techniques are not as accessible as tonometry, are much more expensive in comparison to the simple tonometric recordings, and render necessary the presence of well-trained personnel to handle the required equipment.

Following a regression analysis’ concept, in a previous *in-silico* study, Vardoulis et al. (2012) demonstrated that C_*T*_ could be effectively derived using only cfPWV. They provided a simple equation that directly relates the cfPWV measurement to C_*T*_. The results hypothesized that solely cfPWV should be sufficient for accurately estimating C_*T*_. Further light upon the significance of including more features to the regression method can be provided by assessing the features’ importance levels. As per the feature importances of our study, indeed cfPWV appeared to be among the most significant parameter for estimating C_*T*_. In order to further verify the necessity of including additional features to cfPWV (namely, cuff BP and crPWV), we predicted C_*T*_ using only cfPWV. Concretely, following a similar approach with Vardoulis et al. yielded a lower prediction precision with an nRMSE = 12.4%, a zero bias, while LoA were reported to be ± 0.64 mL/mmHg. This error is approximately 2 times higher than the error provided by the ML model of this study (6.26% from ANN estimator). Although the cfPWV-based estimator performed adequately, we may deduce that inclusion of both cfPWV and crPWV improves the precision in C_*T*_ estimation. Importantly, this apparently slight improvement might be rather necessary when performing the analysis on an *in-vivo* population. Yet, the regression analysis, which uses both PWV values might provide a more clinically relevant estimation of C_*T*_, as it combines both a proximal and a distal approximation of arterial stiffness, and thus a more complete description of the arterial tree’s elasticity.

This study further explored and quantified the importance of every input for the predictive performance. Diastolic pressure had the most significant contribution to the estimations. This is to be expected given that both Z_*ao*_ and C_*T*_ are strong determinants of mean pressure and by extension the brDBP. Furthermore, most of the arterial compliance is contained in the aorta. This could explain why the permutation importance of cfPWV (namely, aortic PWV) was found to be slightly higher compared to crPWV (Table 5). Yet, the two PWV inputs presented similar, high importance levels. It is highly possible that the bulk of the needed information for Z_*ao*_ and C_*T*_ is contained in the common arterial path included in both cfPWV and crPWV; namely, the arterial segments which are closer to the aorta. Hence, the inclusion of both is important to detect and reveal this joint information. Attention must be paid to the fact that this study uses synthetic data produced by a simulator and, hence, there is a direct deterministic relation between the input and the outputs. This relation may lead to an increased accuracy in the predictions. The results regarding the importance of each feature would be of benefit if they are considered in a qualitative way.

We additionally evaluated the models’ sensitivity to the inputs by training and testing the models using different sets of features. Given that the HR has been shown to have a pressure-independent impact on PWV (Bikia et al., 2020c), we decided to include it in our experiments in order to consider its independent contribution and to enhance the clinical relevance of our results. Nevertheless, it was shown that exclusion of HR from the input vector did not harm the accuracy. Moreover, both RFR and ANN performed adequately when brSBP and brDBP were replaced by MAP. However, when only the PWV values were fed to the models, the precision was deteriorated for both Z_*ao*_ and C_*T*_. This may be explained due to the BP-dependency of PWV which has been shown to have implications for the clinical use of arterial stiffness measurements, both in risk classification and in treatment monitoring of individual patients (Spronck et al., 2015).

Recent advancements in Artificial Intelligence (AI) have led to new research possibilities and methodologies for novel cardiovascular modeling and predictive tools for clinical use (Ramesh et al., 2004). The present study is in line with this direction that introduces AI to the field of clinical medicine. There have been several novel works toward this path, including estimation of PWV or central BP (Greve et al., 2016; Xiao et al., 2017; Tavallali et al., 2018). ML modeling allows for enhancing monitoring of vascular biomarkers via the analysis of complex datasets, signals and/or images. The availability of large clinical datasets and powerful computing systems further encourage the application of ML-based concepts. In addition, nowadays, vascular parameters or arterial pulse signals can also be obtained using unobtrusive devices such as smartphones and smartwatches, providing a plethora of available data.

The main limitation of the present study is that the data used in the analysis have been derived from a computer simulator rather than a real human population. A ML model which is trained/tested using *in-silico* data, it is likely that it will not be capable of making accurate predictions for a real patient. Yet, the *in-silico* data allow us for performing an initial validation of the proposed methodology, whose results will allow to proceed with the clinical validation. Previous works have used a similar approach to validate ML-based techniques using virtual patients when real clinical data were not available (Huttunen et al., 2019; Bikia et al., 2020b, 2021; Huttunen et al., 2020). Hence, the present study proposes the methodology rather than the model *per se*. *Stricto sensu*, based on the findings from an *in-silico* population, we may only deduce that the proposed ML-based methodology could also work using real human data for both the training and testing procedures. The latter hypothesis remains to be verified *in vivo*. Future work will be done toward this direction. Nevertheless, synthetic data can sufficiently simulate the content of the real clinical measurements, while they allow for controlling the distribution of rare but relevant conditions or events. It is also to be mentioned that the *in-silico* data allow us for appraising this concept using the actual Z_*ao*_ and C_*T*_, which are derived analytically from the computer simulator and would not have been available *in vivo*. Future work should also include validation of the method on populations with pathologies or special populations.

Finally, a major consideration with respect to the application of ML in Healthcare is generalizability, i.e., the ability of a model to predict accurately on data sources which are not included in the dataset of the specific study. Dexter et al. (2020) demonstrated that studies showing high-performance ML models may not perform well when applied to data from other holdout systems. Each modeling strategy is limited by assumptions and data collection is dependent on several factors, including clinical context, local factors (e.g., physician preferences, local care standards), medication selection or other clinical decisions which influence the model development (Shameer et al., 2018). Therefore, direct validation of a ML algorithm to a new dataset should not assume model’s strong performance on every other dataset; even when the model is trained using real clinical data. The above limitations underline the need to consider more inclusive training approaches for ML models which could encourage the practical application of ML in Healthcare.

## Conclusion

This paper introduces a non-invasive simple-to-use estimator for two valuable hemodynamic quantities, namely, the Z_*ao*_ and C_*T*_. The proposed approach incorporates cuff blood pressure and regional PWV data, along with a versatile and scalable ML pipeline. Our findings provide evidence that data related to regional arterial stiffness can be rather informative for obtaining a global description of arterial elasticity. Further validation of the proposed methodology on a large human cohort remains to be conducted. Upon successful clinical validation, this framework may provide a reliable method to inform the clinicians about arterial stiffness, leading to an improved diagnosis and patients’ treatment management.

## Data Availability Statement

The raw data supporting the conclusions of this article are available in the Supplementary Material.

## Author Contributions

VB and NS conceived and designed the research. VB analyzed the data and trained, tested the machine learning models, generated all figures and tables, and drafted the manuscript. All authors discussed the results and edited the manuscript.

## Conflict of Interest

The authors declare that the research was conducted in the absence of any commercial or financial relationships that could be construed as a potential conflict of interest.
