# Corrigendum: Determination of aortic characteristic impedance and total arterial compliance from regional pulse wave velocities using machine learning: an *in silico* study

**DOI:** 10.3389/fbioe.2024.1345502

**Published:** 2024-03-08

**Authors:** Vasiliki Bikia, Georgios Rovas, Stamatia Pagoulatou, Nikolaos Stergiopulos

**Affiliations:** Laboratory of Hemodynamics and Cardiovascular Technology, Institute of Bioengineering, Swiss Federal Institute of Technology, Lausanne, Switzerland

**Keywords:** non-invasive monitoring, aorta, arterial stiffness, vascular aging, machine learning

In the published article, there was an error. During proof writing, the reference numbering (namely, (13) as provided by the authors) was not replaced. As a result, the formulas in the section indicated below were followed by “(13)” which does not symbolize anything in the paper.

A correction has been made to **Materials and methods**, Comparison to Prior Art, Paragraph 1 (bullet points 1 and 2) to remove “(13)”.

This sentence previously stated:

“1. *Time-derivative peaks method*: Z_ao_ = P’_max_/Q’_max_ (13), where P’_max_ and Q’_max_ are the maximum values of the pressure and flow time derivatives, respectively.

2. *Peak flow method*: Z_ao_ = (P_Qmax_–aDBP)/Q_max_ (13), where aDBP is the aortic DBP, Q_max_ is the maximum flow value, and P_Qmax_ is the aortic pressure magnitude at the maximum flow value.”

The corrected sentence appears below:

“1. *Time-derivative peaks method*: Z_ao_ = P’_max_/Q’_max_, where P’_max_ and Q’_max_ are the maximum values of the pressure and flow time derivatives, respectively.

2. *Peak flow method*: Z_ao_ = (P_Qmax_–aDBP)/Q_max_, where aDBP is the aortic DBP, Q_max_ is the maximum flow value, and P_Qmax_ is the aortic pressure magnitude at the maximum flow value.”

The authors apologize for this error and state that this does not change the scientific conclusions of the article in any way. The original article has been updated.

